# Clinical manifestations and MRI features of Danon disease: a case series

**DOI:** 10.1186/s12872-023-03356-y

**Published:** 2023-08-11

**Authors:** Yang Zhang, Ren Zhao, Yushan Yuan, Yongqiang Yu, Bin Liu, Xiaohu Li

**Affiliations:** 1 Department of Radiology, Fuyang People’s Hospital, Fuyang, 236000 Anhui China; 2 Department of Cardiology, The First Affiliated Hospital of Anhui Medical University, Hefei, 230022 Anhui China; 3 Department of Radiology, The First Affiliated Hospital of Anhui Medical University, Hefei, 230022 Anhui China; 4 Research Center of Clinical Medical Imaging, Hefei, 230032 Anhui Province China; 5 Department of Radiology, First Affiliated Hospital of Anhui Medical University, No.218 Jixi Road, Hefei, 230022 Anhui China

**Keywords:** Danon disease, Magnetic resonance imaging, Extracellular volume fraction, T1 mapping

## Abstract

**Background:**

Danon disease (DD) is an exceptionally uncommon X-linked dominant lysosomal glycogen storage disorder characterized by pronounced ventricular hypertrophy and cardiac insufficiency. The timely identification of cardiac impairment in individuals with DD holds significant clinical importance.

**Case presentation:**

We present a case of Danon Disease in a three-generation pedigree from Anhui Province, China. Clinical features and laboratory data were collected and analyzed for a 16-year-old male proband (III-1) and two affected female family members (II-2 and II-3). The proband exhibited Wolf-Parkinson-White syndrome, hypertrophic cardiomyopathy, abnormal cognitive function, and muscle weakness. Gene sequencing confirmed a mutation (c.963G > A) in the LAMP-2 gene.

**Conclusion:**

Patients with DD may present both dilated and hypertrophic cardiomyopathy. Comprehensive myocardial tissue characterization by MRI plays a key role in the diagnosis of the disease.

**Supplementary Information:**

The online version contains supplementary material available at 10.1186/s12872-023-03356-y.

## Background

Danon disease (DD) is a rare X-linked dominant lysosomal glycogen storage disease that manifests as severe ventricular hypertrophy and heart failure [1]. Currently, detection of mutations in the lysosomal-associated membrane protein-2 (LAMP-2) gene is the gold standard for DD diagnosis [2], and endocardial biopsy is also a critical method for diagnosing it. If a genetic test is unavailable, endocardial biopsy by electron microscopy combined with clinical triad can be used for clinical diagnosis.

Cardiovascular magnetic resonance (CMR) is a rapidly evolving non-invasive imaging modality offering comprehensive, multiparametric assessment of cardiac structure and function in various clinical situations. CMR does not expose patients to ionizing radiation, and its high spatial resolution enables detailed myocardial tissue characterization [3]. Late gadolinium enhancement (LGE) CMR imaging is the most sensitive imaging technique for identifying the extent of myocardial infarction or assessing residual myocardial viability. Myocardial T1 mapping is a non-invasive technology to assess the extracellular volume fraction (ECV), which reflects the degree of diffuse myocardial fibrosis by measuring myocardial and blood T1 relaxation time before and after contrast enhancement [4].

Typical DD treatments aimed at preventing sudden cardiac death and alleviate symptoms, and heart transplantation is still its sole radical treatment. In addition, in vivo study using LAMP-2 KO mice, Manso et al. attempted to rescue LAMP-2B deficiency via recombinant adeno-associated virus 9 (AAV9).The survival rate in older mice treated with gene therapy treatment was evidently improved, and a phase 1 clinical trial is currently underway to assess the safety and toxicity of this gene therapy product in human DD [5]. Small molecule-based approaches to modify the autophagy process have also been shown to have therapeutic effects in several studies [6]. Due to the low prevalence of DD, most previous studies are case reports, and multiple cases in one family are rare. The diagnostic value of CMR in DD and the cardiac efficacy of various treatments still needs to be explored. In this study, we describe the CMR characteristics of three cases of DD in a Chinese family, aiming to explore the value of multi-parameter MR characteristics in DD diagnosis and follow-up after treatment.

## Case presentation

### Clinical features and associated laboratory data

The three generations of the proband’s immediate family members were investigated. Clinical data, including sex, age, symptoms, electrocardiography, extracardiac presentation, and laboratory data, were collected and analysed (Table 1). Their pedigree was obtained from hospital records in Anhui Province, China. Gene sequencing verified that the proband carried the mutation c.963G > A in the LAMP-2 gene.

**Table 1:** Baseline characteristics, clinical presentation, and assisted testing of patients with DD

Danon patients	Proband (III-1)	Mother of the proband (II-2)	Aunt of the proband (II-3)
**Sex/age (years)**	Male/16	Female/37	Female/39
**Clinical ** **presentation**	Shortness of breath	Shortness of breath	Shortness of breath and palpitation
**NYHA class**	II	III	II
**ECG**	HVLV and WPW	Ventricular premature beat and LBBB	Ventricular premature beat
**Skeletal myopathy**	Yes	No	No
**Intellectual retardation**	Yes	No	No
**NT-pro-BNP (pg/mL)**	/	564.94↑	3450↑
**AST (U/L)**	332.1↑	144↑	30.1
**ALT (U/L)**	243.7↑	153↑	13.2
**LDH (U/L)**	943.9↑	962↑	259.8
**CK (U/L)**	1393.1↑	66	49.7
**CREA (U/L)**	50.5	59.1	57.8
**BUN (U/L)**	7	4.6	4.6

NYHA, New York Heart Association; ECG, electrocardiogram; HVLV, high-voltage left ventricle; WPW, Wolf-Parkinson-White syndrome; pro-BNP, pro-B-type natriuretic peptide; AST, aspartate transaminase; ALT, alanine aminotransferase; LDH, lactate dehydrogenase; CK, creatine kinase; CREA, creatinine; BUN, blood urea nitrogen

↑: elevation compared to reference range

The proband (III-1) was a 16-year-old male who had been previously reported [7]. He was previously diagnosed with Wolf-Parkinson-White (WPW) syndrome and hypertrophic cardiomyopathy (HCM). Catheter ablation was performed a year earlier, but the effect was not satisfactory. The electrocardiogram showed a high voltage in the QRS complexes and a short PR with a delta wave, indicating WPW syndrome and left ventricular hypertrophy. He also displayed abnormal cognitive function and decreased muscle strength.

According to the clinical data, two other patients with DD were found in the family: the proband’s mother (II-2) and aunt (II‐3).

**Fig. 1:**
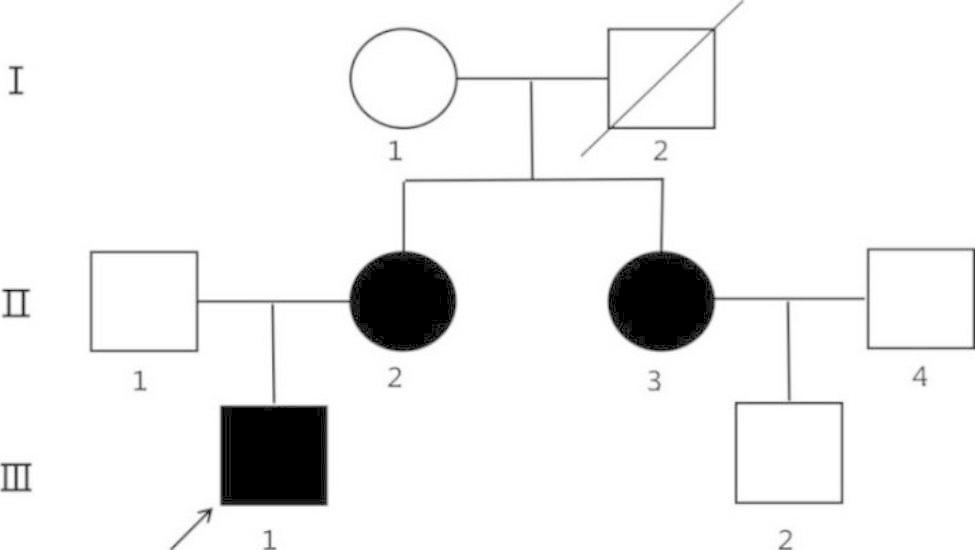
Pedigree of the family in this report. Squares: male individuals; circles: female individuals; slashes: deceased individuals; filled black shapes: affected patients; arrow: proband

The father of patients II-2 and II-3 had died 20 years earlier. The other family members did not show exercise intolerance or psychomotor development. Their electrocardiogram and echocardiogram were normal. The pedigree of the proband (III-1) and other family members is shown in Fig. 1. The clinical presentation and laboratory results from the three patients in this family are summarized in Table 1.

### CMR characteristics

**Fig. 2:**
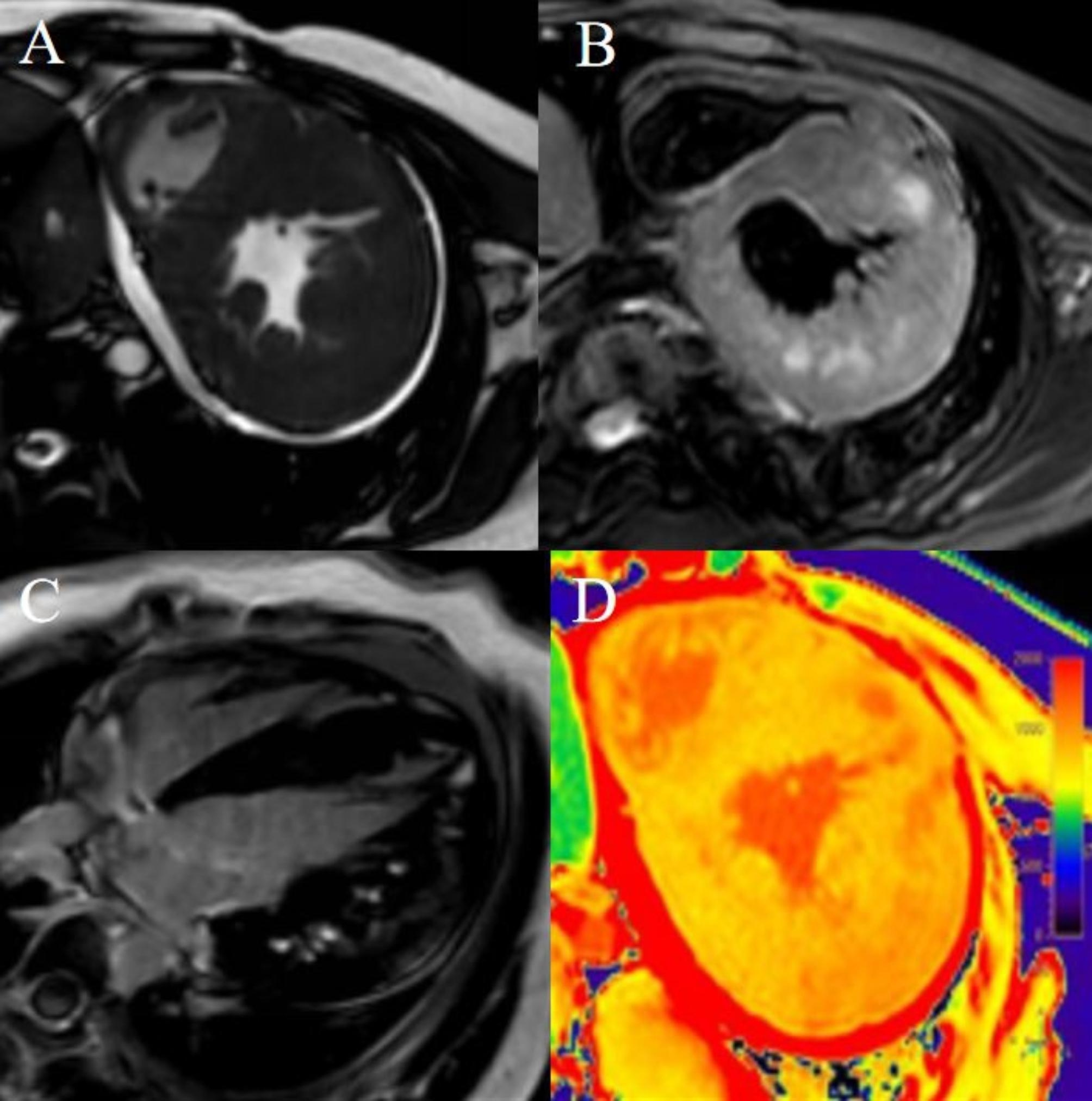
A 16-year-old male (proband, III-1) had exertional dyspnoea for 2 years along with intellectual disability. He was genetically diagnosed with DD (mutation site: c.963G > A). Cine images show concentric hypertrophic cardiomyopathy. The right ventricular wall is thickened (**A**), and the hyper-T2 signal manifests at the left ventricular free wall (**B**). LGE shows involvement at the left ventricular free wall (**C**). The native T1 value of the left ventricular free wall is high (**D**)

**Fig. 3:**
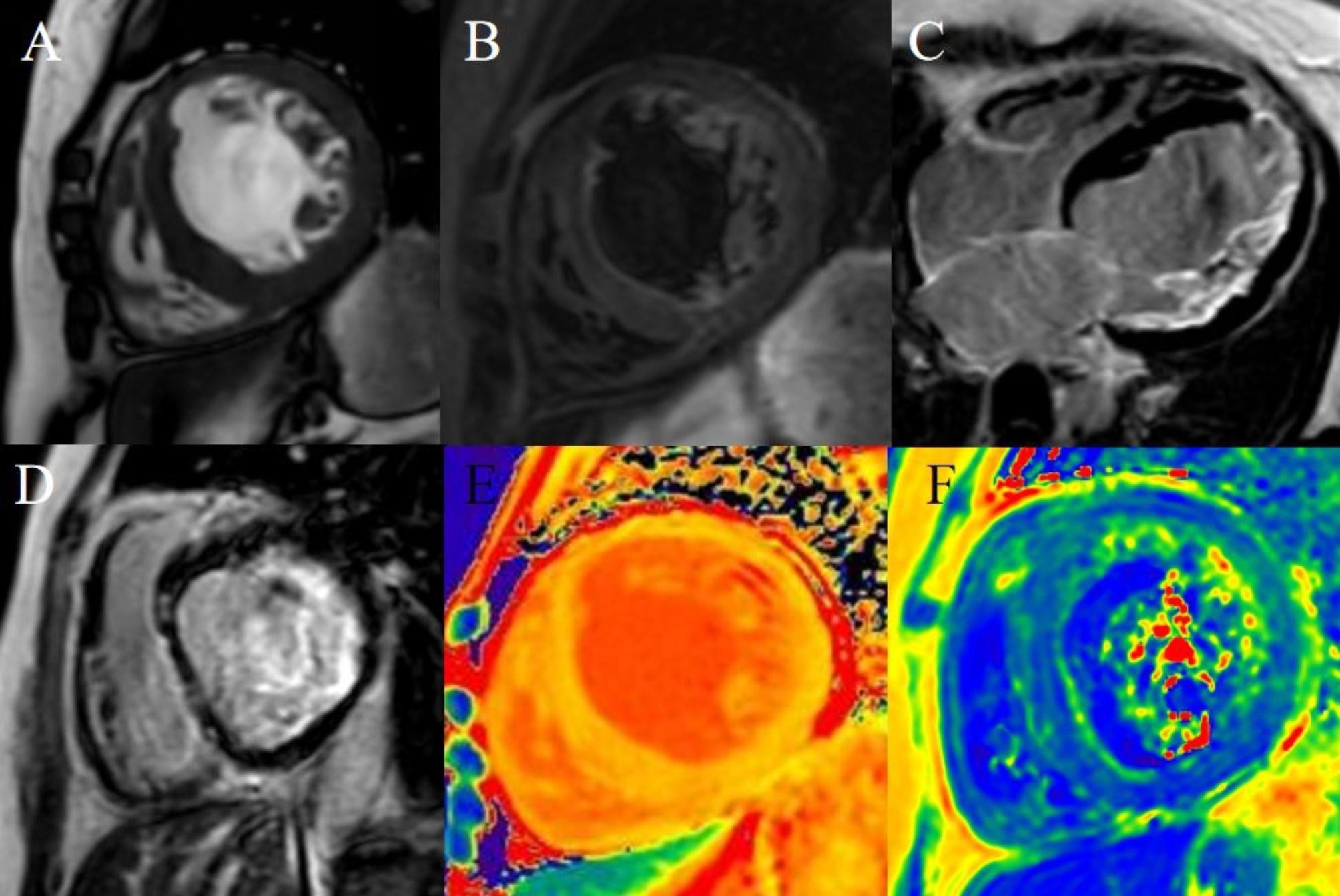
A 37-year-old female (II-2), the proband’s mother, suffered from shortness of breath for > 3 months. Cine images show symmetric hypertrophic cardiomyopathy with systolic dysfunction in the left ventricle (**A**). The left ventricular mass was 187 g, LVEF was 13%, and the EDV and ESV were 221ml and 192ml, respectively. There was no obvious hyperintensity on T2 fat-saturated images (**B**). LGE shows extensive subendocardial involvement at the left ventricular free wall (**C, D**). The native T1 values of the left ventricular myocardium were elevated (**E**). The T2 values of the left ventricular free wall were elevated (**F**)

**Fig. 4:**
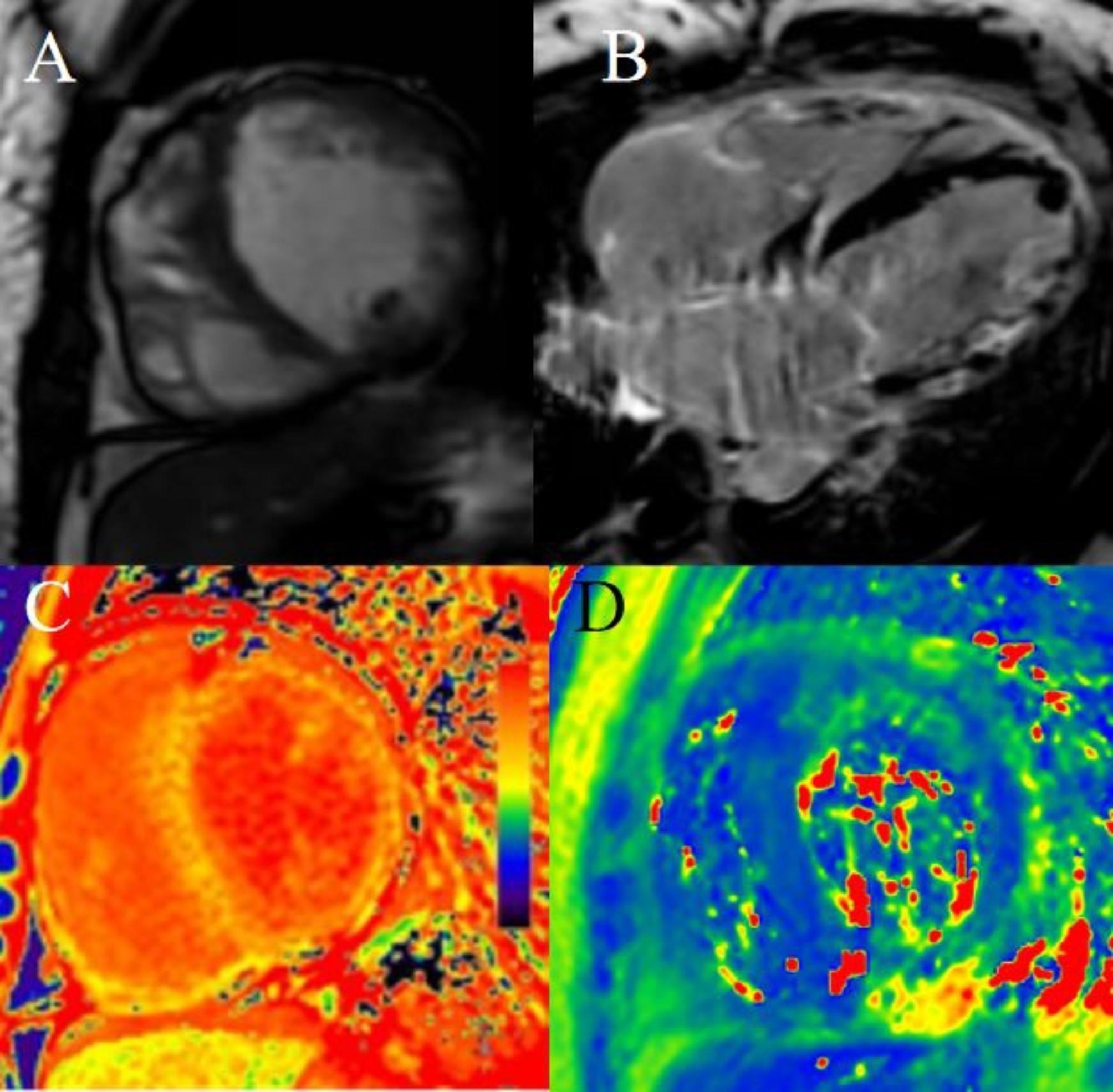
A 39-year-old female (II-3), the proband’s aunt, suffered from shortness of breath and palpitation. Cine images show dilated cardiomyopathy with biventricular systolic dysfunction (**A**). The LV mass was 84.9 g, LVEF was 17%, and the EDV and ESV were 145ml and 120ml, respectively. An apical thrombus can be seen in the left ventricle. LGE shows basal-middle septum sparing and an apex with extensive transmural feather-like involvement at the left ventricular free wall (**B**). The native T1 values of the left ventricular myocardium were elevated (**C**). The T2 values of the left ventricular free wall were elevated (**D**)

Table 2 shows the CMR features of patients in this family. Two patients presented HCM phenotypes, and one showed a dilated cardiomyopathy (DCM) phenotype. LGE images showed that all patients had basal-mid septum sparing and free wall involvement. The native T1 and ECV values of the left ventricular myocardium were elevated. The T2 values of the left ventricular free wall were high, while those in the septal wall were in the normal range in all three patients. (Table 3). Three patients showed intramyocardial patchy LGE, subintimal LGE, and feather-like transmural LGE (Figs. 2, 3 and 4). The T1 and ECV values of the left ventricular myocardium in all the DD patients were elevated, especially in the corresponding LGE region. The T2 values of the septal wall were within the normal range, while those of the free wall were elevated (Figs. 2, 3 and 4).

**Table 2:** Phenotypes and CMR characteristics of DD

DD patients	Left ventricle phenotype and patten			Left ventricle tissue features				
	Normal	HCM	DCM	LGE Presence	Basal-mid-septum sparing	Free-wall involvement	Subendocardial involvement	Hyperintensity T2 signal
Proband (III-1)	No	Yes	No	Yes	Yes	Yes	No	Yes
Mother of the proband (II-2)	No	Yes	No	Yes	Yes	Yes	Yes	No
Aunt of the proband (II-3)	No	No	Yes	Yes	Yes	Yes	Yes	No

**Table 3:** T2, native T1 and ECV values of the left ventricular myocardium

DD patients	Free wall			Ventricular septum		
	T2	Native T1	ECV	T2	Native T1	ECV
Proband (III-1)	59.06 ms	1456 ms	35.40%	45.95 ms	1297 ms	27.80%
Mother of the proband (II-2)	56.13 ms	1406 ms	39.10%	44.85 ms	1367 ms	33.70%
Aunt of the proband (II-3)	61.44 ms	1529 ms	40.30%	49.15 ms	1432 ms	30.60%

## Discussion and conclusion

DD is rare, and the disease is inherited as an X-linked dominant trait. The primary deficiency of LAMP-2 causes disruption of autophagy, leading to impaired fusion of lysosomes. Patients often present with clinically diseased heart muscle (cardiomyopathy), weakness of body muscles (skeletal myopathy), and neurobehavioural problems [8]. The myopathy is usually more severe in men than in women. In one cohort of 38 DD patients, all patients had cardiomyopathy, 18/20 male patients (90%) and 6/18 female patients (33%) had skeletal myopathy, and 14/20 male patients (70%) and 1/18 patients (6%) presented neurobehavioural problems [9]. In our group, the male patient (III-1) suffered from mild muscle weakness and cognitive dysfunction, while the female patients (II-2 and II-3) did not.

DD can also affect other systems. LAMP-2 is expressed in the retinal pigment epithelium [10], and approximately 60–70% of patients have colour vision disorders, macular degeneration, and other symptoms [11]. Some patients may present hepatosplenomegaly, abnormal renal function, and even autism [12]. In the present study, 2 out of 3 patients had severe liver function abnormalities. Therefore, we speculate that liver enzyme abnormalities in DD may not be uncommon and may show no differences between sexes.

Although DD is a multisystemic disease, heart failure and arrhythmia are the leading causes of morbidity and mortality [13]. Wei et al. [14] described the cardiac MRI findings in a cohort of 16 patients with DD, which is the largest cardiac MRI case series of patients with DD published to date. All male patients (n = 13) showed myocardial hypertrophy, of which symmetric HCM was the most common type (56%), followed by asymmetric HCM (38%). All the female patients presented myocardial hypertrophy or ventricular enlargement. These findings differed from those of Taylor et al. [15] in a group of patients with familial DD, who showed that 3/8 (37.5%) male patients had DCM, 4/8 (50%) had HCM, and a 1-year-old patient did not show any cardiac manifestations.

In previous studies, DD patients have shown reduced left ventricular function, but the decreases in male and all the female patients have been different. Wei et al. [14] found that the average ejection fraction of male patients was lower than that of females (30% vs. 65%). According to Lotan et al. [16], 40% of men and 59% of women showed decreased left ventricular systolic function (the LVEF of men and women was 34 ± 11% and 28 ± 13%, respectively).

In our cohort, the male patient presented the HCM phenotype with normal ejection fraction, and one female patient presented the HCM phenotype, but the other presented DCM with left ventricular dilation as well significantly reduced ejection fraction. Some studies have demonstrated that decreased LVEF or dilated left ventricle might be a manifestation of advanced HCM. As seen in patient II-3, the left ventricular apical thrombus might be associated with reduced LVEF.

In the study by Wei et al. [14], LGE patterns were observed in almost all patients with diffuse distribution. The characteristic LGE features included free wall involvement (94%) and basal-middle septum sparing (88%) [14]. However, in the study by Rigolli et al. [17], the incidence of LGE was lower (73%), which might be due to the older age of the patients, the higher the proportion of male patients, and the more severe disease.

Zhou et al. [18] reported that the native T1 value of a patient with DD was low, but in the study by Wei et al. [14], elevated values were found. Due to the intracellular glycogen storage of DD, the T1 value can be reduced in the early stage [19], but the T1 value of the patients at the later stage in the present study increased due to extracellular fibrosis. In our cohort, all three patients underwent T1 and T2 mapping examinations and further ECV calculation.

This study had some limitations. Although we reported three cases of danon disease in one family, cases were from only one hospital and the number was still small. Second, although we have recommended the proband to make an appointment for heart transplantation, they refused to follow the suggestion for economic reasons or the lack of understanding on severity of the disease. We will continue to follow up with the patients to further evaluate the progress and efficacy of the disease.

In summary, we report three cases of DD in a Chinese family, covering their clinical manifestations and CMR characteristics, including sex differences. Since DD is rare, it may be misdiagnosed as other non-ischaemic heart diseases, such as HCM and DCM. A comprehensive CMR examination might play a critical role in diagnosis and severity and risk factor grading. Hence, CMR LGE analysis and T2 and T1/ECV mapping are key in assessing subtle cardiac dysfunction and myocardial fibrosis in DD patients at an early stage.

### Electronic supplementary material

Below is the link to the electronic supplementary material.

**Additional File 1:** CARE Checklist of information to include when writing a case report

## Data Availability

The datasets used and/or analyzed during the current study are available from the corresponding author on reasonable request.

## Abbreviations

DD: Danon disease
LAMP-2: Lysosomal-associated membrane protein-2
CMR: Cardiovascular magnetic resonance
LGE: Late gadolinium enhancement
ECV: Extracellular volume fraction
AAV9: Adeno-associated virus 9
WPW: Wolf-parkinson-white
HCM: Hypertrophic cardiomyopathy
DCM: Dilated cardiomyopathy
